# A Phase-Divided MSPC Approach for Wing Strain Data Characterization and Anomaly Identification

**DOI:** 10.3390/s26196289

**Published:** 2026-10-04

**Authors:** Cuicui Du, Tao Ge

**Affiliations:** School of Electrical & Information Engineering, Jiangsu University of Technology, Changzhou 213001, China; g19852682228t@163.com

**Keywords:** anomaly identification, FBG strain data, multivariate statistical process control (MSPC), strain data, time-domain features, wing structure

## Abstract

The effective characterization and anomaly identification of flight strain data are critical to aircraft structural health monitoring. To address the challenges of large-volume wing strain data and the limitations of univariate methods in capturing synergistic variations among multiple features, this paper proposes a phase-divided multivariate statistical process control (MSPC) approach for wing strain data characterization and anomaly identification. Focusing on the rear spar flange of a specific aircraft wing, independent multivariate control baselines are established for the takeoff, cruise, and landing phases. A six-dimensional feature vector is constructed using peak value, root mean square, standard deviation, peak-to-peak value, kurtosis, and skewness to quantitatively characterize the strain data of the wing structure across different flight phases. After feature standardization, a Hotelling’s multivariate statistical control model is developed to identify anomalies in structural strain states. Based on 30 normal sorties, the upper control limit is determined as UCL = 26.58 at a 99% confidence level. Anomaly diagnosis is performed on six test sorties using *T*^2^ control charts and contribution plots, successfully identifying three sorties with anomalous strain patterns and tracing their dominant feature parameters. The results demonstrate that the proposed method can effectively characterize the strain features of wing structures in different flight phases and identify anomalous strain patterns and their dominant contributing factors. Comparisons with a univariate threshold method further confirm the advantages of the proposed method in capturing synergistic anomalies and providing cause tracing.

## 1. Introduction

In aircraft structural health monitoring, sensor networks are typically mounted on key components of aircraft (e.g., wings, tails, etc.) to obtain real-time response data [1,2,3]. The wing, which is regarded as the primary aerodynamic and load-bearing structure of an aircraft, is subjected to significantly different loading patterns during typical flight phases such as takeoff, cruise, and landing. The strain response directly reveals the structural stress characteristics under typical flight phases [4,5]. Therefore, it is essential to accurately characterize the wing strain features across typical flight phases to understand its mechanical response and evaluate structural conditions.

Although accurate strain characterization is essential for understanding mechanical response and evaluating structural states, a single-sortie strain curve reflects only that flight’s amplitude variations, hindering the identification of structural evolution across multiple sorties [6,7]. Strain data grow exponentially with sortie accumulation, making manual comparison inefficient for anomaly detection. Unexpected strain fluctuations or impacts may accelerate fatigue accumulation and compromise flight safety [8,9]. Therefore, a method capable of automatically extracting state information and identifying anomalies from large-scale strain data is urgently needed for reliable wing health monitoring.

In the field of structural health monitoring, time-domain statistical parameters are widely used for feature extraction and state characterization of strain and vibration signals, serving as the statistical basis for establishing baseline references of structural normal states and conducting anomaly identification [10,11,12,13]. For example, in ref. [14], a robust method that fuses one-dimensional local binary pattern features and time-domain statistical features was proposed, which then feeds them into an XG-Boost classifier for anomaly detection, with a feature simplification strategy to enhance efficiency. In ref. [15], the authors proposed a hybrid deep-learning and improved inverse-direct method for in-service load tracking of sensor-limited aircraft, where the deep learning model predicts discrete strains from flight parameters and the inverse-direct approach enhances accuracy using reduced-order aerodynamic modes. In ref. [16], a time-frequency feature fusion method for fault detection was presented in this paper, integrating statistical time-domain features with EMD-ICA and Wigner bispectrum-based frequency-domain characteristics to overcome the limitations of downsampling-induced data degradation. Zhang et al. [17] proposed a hybrid physics-based and data-driven framework using a Windowed Sparse-Attention Transformer for tool remaining useful life prediction under time-varying processing parameters, demonstrating the potential of deep learning in small-sample scenarios. While these studies were originally developed for non-aerospace applications (e.g., bridges and mechanical systems), they provide the methodological foundation for multivariate statistical process control and anomaly detection, and their underlying principles are directly transferable to aerospace structural health monitoring. However, despite the valuable progress made by the aforementioned investigations in structural condition monitoring and anomaly identification, several inherent limitations remain: these methods lack anomaly cause tracing and exhibit strong configuration dependence with weak generalization [18,19,20,21,22].

In response to the above limitations, this study proposes a phase-divided multivariate statistical process control (MSPC) approach for wing strain characterization and anomaly identification. Unlike conventional phase-wise MSPC and Hotelling’s T^2^ monitoring, the proposed method establishes independent control baselines for the takeoff, cruise, and landing phases of flight strain data and integrates contribution plots for anomaly cause tracing, which has not been systematically investigated for aircraft wing structures. The flight process of the wing is divided into three typical stages—takeoff, cruise, and landing phases—and six time-domain features are extracted to characterize the strain response from multiple perspectives. A Hotelling’s T^2^ control chart is established to detect anomalies, while contribution plots are employed to trace the dominant features responsible for detected deviations.

## 2. Wing Structure and Flight Strain Monitoring Data Selection

### 2.1. Wing Structure

**Figure 1:**
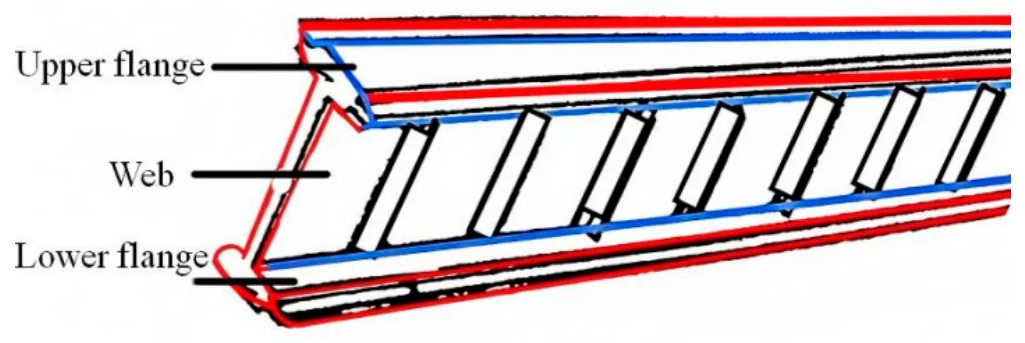
Schematic diagram of the wing spar structure.

The wing structure mainly consists of components such as skin, spars, ribs, and stringers. As shown in Figure 1, the wing spar, which serves as the principal longitudinal structural member of the wing, consists of webs and flanges and carries all or most of the wing bending moment [23,24].

**Figure 2:**
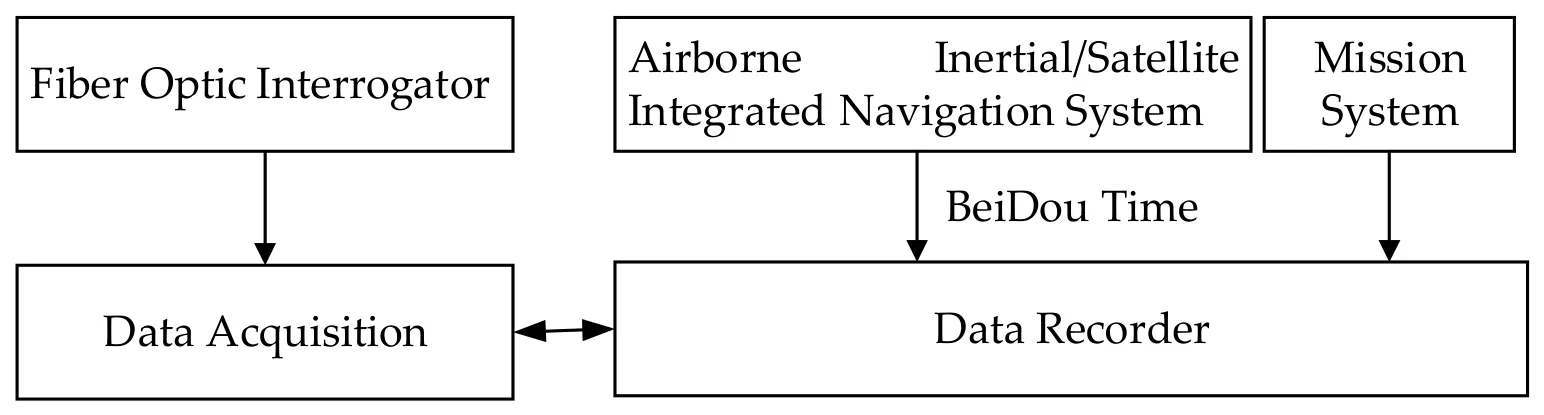
Functional block diagram of the data acquisition unit and data recorder.

In view of the installation environment of the structural components of a certain aircraft, a substrate-type FBG strain sensor was chosen due to its advantages of being calibratable, highly linear, and having a long service life. In this study, fiber Bragg grating strain sensors are mounted on the rear spar flange to acquire strain response data under different flight states. The data acquisition and recording system is responsible for acquiring and storing strain parameters. The functional block diagram of the data acquisition unit and the data recorder is shown in Figure 2. The data acquisition collects the output data from the fiber optic interrogator, which is then transmitted to the data recorder via a communication cable and stored in the recorder. The data recorder can receive BeiDou time information provided by the aircraft’s airborne inertial/satellite integrated navigation system, thereby achieving synchronization with the aircraft flight time. Table 1 lists the relevant information on the FBG sensors and the data acquisition and recording system.

The substrate-type FBG strain sensor and fiber optic interrogator were purchased from Beijing Institute of Telemetry, Beijing, China. The data acquisition unit (XMA) and data recorder (MDR) were purchased from ZDS, France.

### 2.2. Division of Different Flight Phases

**Figure 3:**
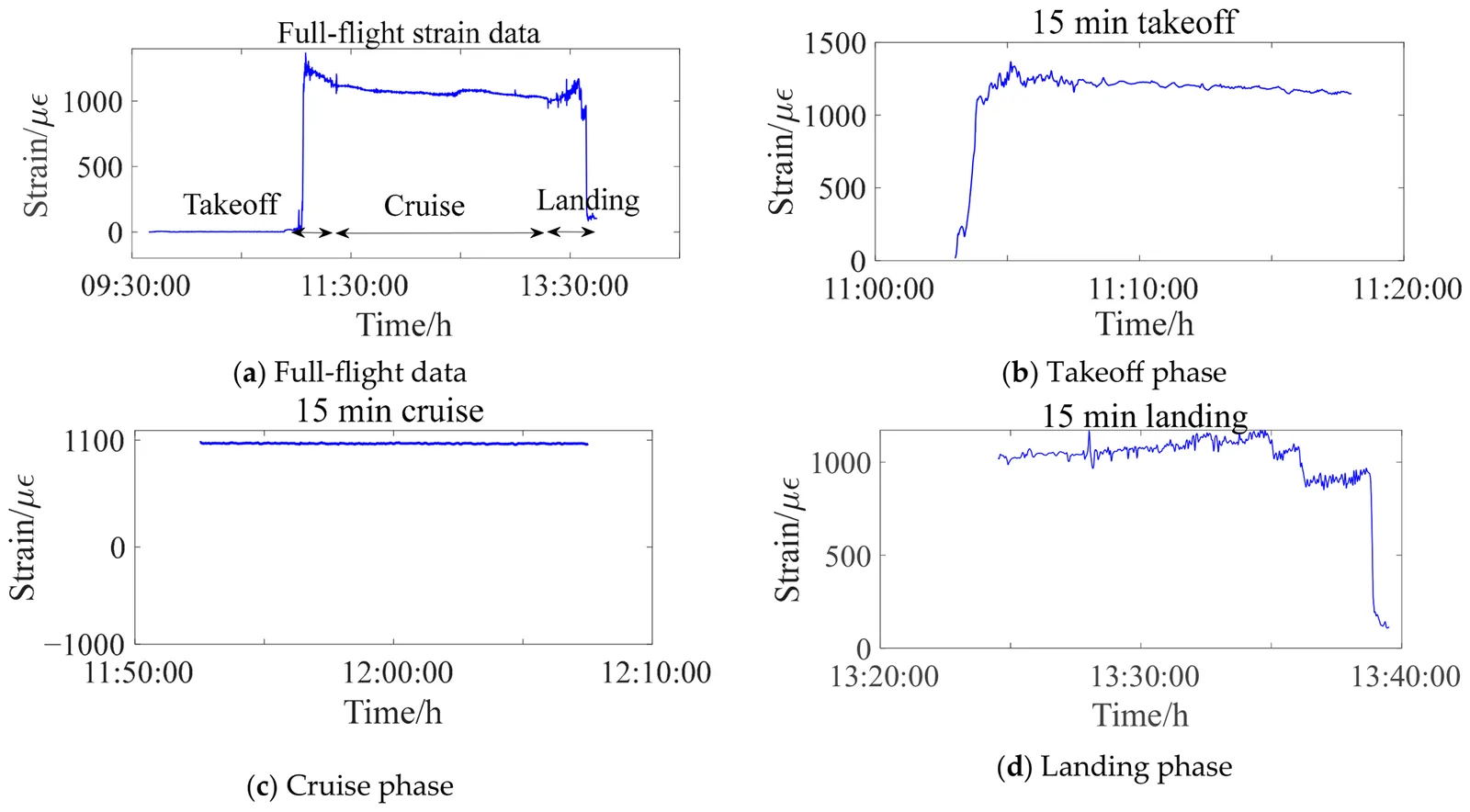
Strain curves of the wing rear spar flange from a representative flight sortie.

The takeoff, cruise, and landing phases of this aircraft type last approximately 15 min, 0.5–2 h, and 15 min, respectively. Given the variable duration of the cruise phase (0.5–2 h) and its susceptibility to factors such as maneuvers and turbulence, exclusive reliance on a fixed algorithm may lead to misclassification. Consequently, according to the flight time recorded by the aircraft data acquisition and recording system, three flight phases—15 min takeoff, 15 min cruise, and 15 min landing—were delineated and manually segmented. Owing to the total flight duration and the number of measurement points, the volume of strain data from a single sortie is considerable. Figure 3 presents the full-flight strain curve of the wing rear spar flange in a representative sortie, along with the corresponding 15 min strain curves for the takeoff, cruise, and landing phases. It is noted that the strain curves shown in Figure 3 were obtained after preprocessing. The preprocessing mainly included temperature compensation and denoising to remove thermal effects and measurement noise.

As shown in Figure 3, a strain jump of approximately 1159 με occurs at the moment of liftoff, followed by a gradual decrease during the climb phase. During the cruise phase, the strain fluctuates steadily around 1025 με. In the landing phase, the absolute value of strain decreases continuously, with a significant jump occurring upon touchdown due to the impact load. The results show that the strain responses of the wing rear spar flange differ significantly across flight phases. Takeoff and landing are characterized by large-amplitude transient impacts, while cruise features high-energy steady-state fluctuations. However, the strain curve of a single sortie can only reflect the time-domain amplitude variations in that particular flight and is insufficient for revealing the evolutionary patterns of structural states across multiple sorties. Therefore, it is necessary to establish a statistical baseline based on strain data from multiple flight sorties, characterize the strain variation patterns across different flight phases through quantitative features, and develop an anomaly identification method to enable effective monitoring of structural states.

## 3. Phase-Divided Approach for Wing Structural Strain Data Characterization and Anomaly Identification

**Figure 4:**
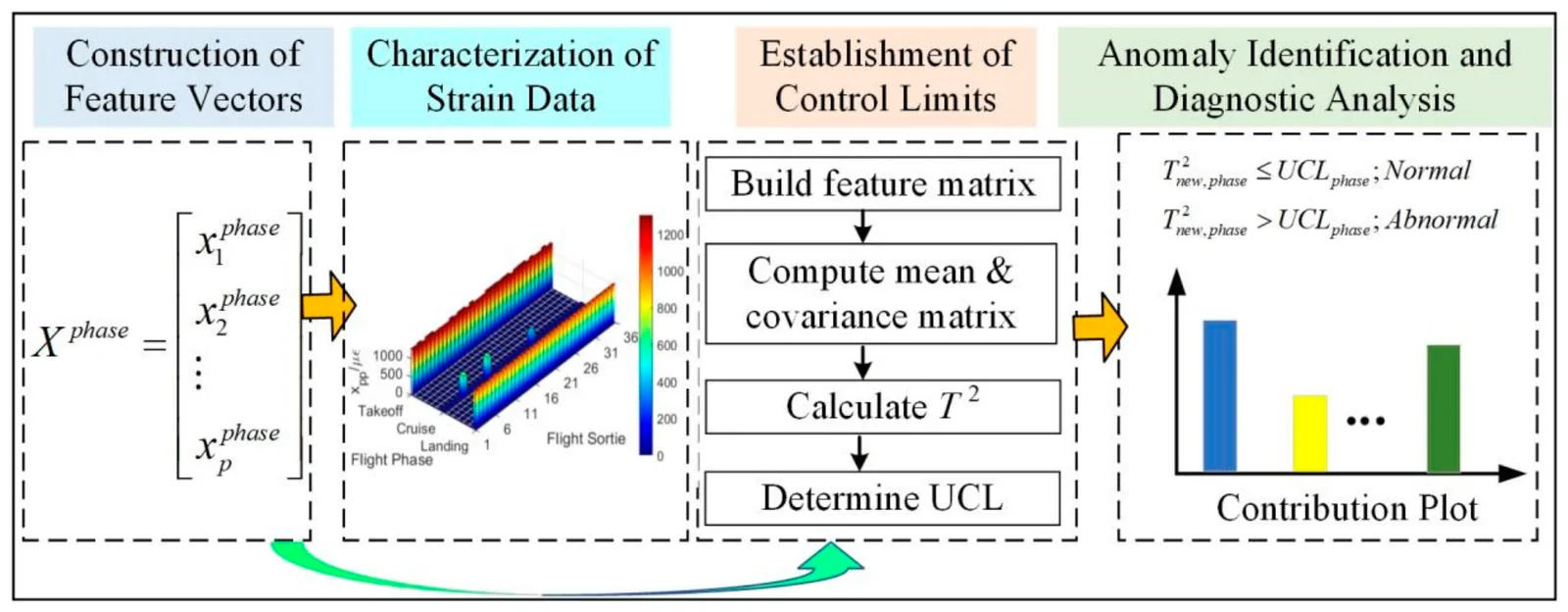
Flowchart of wing structural strain state anomaly diagnosis based on phase-divided MSPC.

To reveal the strain variation patterns of the wing rear spar flange across different flight phases, and to achieve accurate identification of anomalous strain states and localization of anomalous flight phases, this paper proposes a strain data characterization and anomaly identification method based on phase-divided MSPC. As depicted in Figure 4, this framework comprises five core components: feature vector construction, strain data characterization, control limit establishment, anomaly identification and diagnostic analysis, forming a complete mechanism for strain data characterization and anomaly diagnosis of wing structures.

During the takeoff, cruise, and landing phases, time-domain features are extracted to construct multi-dimensional feature vectors so as to characterize the strain patterns of the wing under different flight states. Subsequently, the feature matrix for normal flight samples is constructed, with the sample mean vector, covariance matrix, and T^2^ statistic subsequently derived from it. The upper control limit (UCL) is thereby determined and subsequently applied to conduct anomaly diagnosis on the strain states of the test flight sorties across each flight phase.

### 3.1. Feature Parameter Selection and Strain Data Characterization

To comprehensively obtain the strain variation patterns of the aircraft wing under different flight phases, six parameters—peak value, root mean square, standard deviation, peak-to-peak value, kurtosis, and skewness—are selected to construct the feature vector, as defined in Table 2.

The peak value $x_{p}$ directly reflects the maximum transient strain amplitude experienced by the wing. The root mean square $x_{rms}$ reflects the average energy level of the signal and characterizes the mean loading state of the structure during sustained flight. The σ quantifies the severity of load fluctuations and acts as an effective indicator to distinguish stationary and non-stationary flight states. The $x_{pp}$ reflects the full amplitude range of alternating positive and negative loads experienced by the wing during takeoff and landing. Kurtosis characterizes the impulsiveness of the signal. Under normal conditions, its value is approximately 3; values exceeding 4 typically indicate impulsive vibrations, enabling the identification of turbulence disturbances or structural anomalies [25,26,27]. Skewness reflects the asymmetry of the load distribution and can be used to identify the dominant trend of structural loading. The six-dimensional feature vector described above can characterize the strain features of the wing from multiple dimensions, including amplitude, energy, dispersion, and waveform morphology, thereby providing feature support for structural state assessment and anomaly detection.

### 3.2. Determination of Control Limits

Suppose there are *k* normal flight sorties, with *p* time-domain feature parameters extracted from each sortie in each flight phase, and the set of normal sorties is denoted as $\Omega_{\mathrm{normal}}$. The data of the wing structure in the takeoff, cruise, and landing phases are then processed separately as follows.

(1) Construction of the normal sample feature matrix

A normal sample feature matrix $X_{phase}$ is constructed from the feature vectors of all normal flight sorties of the wing structure across different flight phases:

(1) $$X_{phase}=\left[\begin{array}{l} x_{1,1}^{phase}\;x_{1,2}^{phase}\;\ldots \;x_{1,p}^{phase}\\ x_{2,1}^{phase}\;x_{2,2}^{phase}\;\ldots \;x_{2,p}^{phase}\\ \;\vdots \;\vdots \;\vdots \\ x_{k,1}^{phase}\;x_{k,2}^{phase}\;\ldots \;x_{k,p}^{phase} \end{array}\right]\in {\mathbb{R}}^{k\times p},\;\mathrm{phase}\in \{\;\mathrm{TK},\;\mathrm{CR},\;\mathrm{LD}\}$$

where $x_{j,m}^{phase}$ denotes the value of the m-th feature of the j-th normal sortie, and TK, CR and LD represent the takeoff, cruise, and landing phases, respectively.

(2) Computation of the sample mean vector and standard deviation vector

The mean and standard deviation of each column of the feature matrix $X_{phase}$ are computed to obtain the p-dimensional mean vector ${\bar{x}}^{phase}$ and standard deviation vector $\sigma_{m}^{phase}$:

(2) $${\bar{x}}_{m}^{phase}=\frac{1}{k}\sum_{j}^{k}x_{j,m}^{phase},m=1,2,\cdots ,p$$

(3) $$\sigma_{m}^{phase}=\sqrt{\frac{1}{k-1}\sum_{j=1}^{k}{\left(x_{j,m}^{phase}-{\bar{x}}_{m}^{phase}\right)}^{2}},m=1,2,\cdots ,p$$

(3) Standardization of the feature matrix

To eliminate the influence of dimensional and magnitude differences among features, the feature matrix is standardized using z-score normalization:

(4) $$Z_{j,m}^{phase}=\frac{x_{j,m}^{phase}-{\bar{x}}_{m}^{phase}}{\sigma_{m}^{phase}}$$

where $Z_{j,m}^{phase}$ is the standardized value of the *m*-th feature of the *j*-th normal sortie.

(4) Computation of the sample covariance matrix

The sample covariance matrix $S^{phase}$ is computed based on the standardized feature matrix $Z_{j,m}^{phase}$:

(5) $$S^{phase}=\frac{1}{k-1}\sum_{j=1}^{k}(Z_{j}^{phase}-{\bar{Z}}^{phase}){\left(Z_{j}^{phase}-{\bar{Z}}^{phase}\right)}^{T}$$

where $Z_{j}^{phase}$ denotes the standardized *p*-dimensional feature vector of the *j*-th normal sortie during this phase.

(5) Computation of the *T*^2^ statistic for each normal sortie

For the *j*-th normal sortie, the *T*^2^ statistic in the given flight phase is computed based on the standardized feature vector:

(6) $$T_{j,phase}^{2}={\left(Z_{j}^{phase}-{\bar{Z}}^{phase}\right)}^{T}{\left(S^{phase}\right)}^{-1}(Z_{j}^{phase}-{\bar{Z}}^{phase})$$

$a_{mn}^{phase}$ is denoted as the element in the *m*-th row and *n*-th column of the inverse covariance matrix ${\left(S^{\mathrm{phase}}\right)}^{-1}$. Equation (6) can then be expanded as

(7) $$T_{j,phase}^{2}=\sum_{m=1}^{p}\sum_{n=1}^{p}(Z_{j,m}^{phase}-{\bar{Z}}_{m}^{phase})a_{mn}^{phase}(Z_{j,n}^{phase}-{\bar{Z}}_{n}^{phase})$$

The *T*^2^ statistic is essentially the squared Mahalanobis distance of the *j*-th sample from the normal sample center ${\bar{Z}}^{phase}$ in the *p*-dimensional feature space, reflecting the overall deviation of the sample from the normal baseline.

(6) Determination of the upper control limit *UCL*

According to multivariate statistical process control theory [27], when the process is in a controlled state and the samples follow a multivariate normal distribution, the *T*^2^ statistic follows an *F*-distribution.

The upper control limit $UCL_{phase}$ is given by

(8) $$UCL_{phase}=\frac{p(k-1)}{k-p}\cdot F_{\alpha }(p,k-p)$$

where $F_{\alpha }(p,k-p)$ denotes the upper α-quantile of the *F*-distribution with *p* and *k-p* degrees of freedom.

It is noted that the $UCL_{phase}$ in Equation (8) is intended for Phase II prospective monitoring, i.e., monitoring new observations based on the established normal baseline. Specifically, this control limit is derived for the *T^2^* statistic of individual new observations when the covariance matrix is estimated from a reference sample. It is not applicable to the control limit for subgroup means, which depends on the subgroup size and follows a different formula.

### 3.3. Anomaly Detection and Diagnostic Analysis

For the test flight sorties, the feature vectors are standardized using the mean vector and standard deviation vector established from the normal sorties:

(1) Feature vector of the test sortie

(9) $$X_{\mathrm{new}}^{phase}={\left[x_{\mathrm{new},1}^{phase},\;x_{\mathrm{new},2}^{phase},\;\cdots ,\;x_{\mathrm{new},p}^{phase}\right]}^{T}\in {\mathbb{R}}^{p},\;\mathrm{phase}\in \{\;\mathrm{TK},\;\mathrm{CR},\;\mathrm{LD}\}$$

where $x_{\mathrm{new},m}^{phase}(m=1,2,\cdots ,p)$ denotes the *m*-th feature value of the test sortie in that flight phase.

(2) Standardization of the test sortie feature vector

(10) $$z_{\mathrm{new},m}^{phase}=\frac{x_{new,m}^{phase}-{\bar{x}}_{m}^{phase}}{\sigma_{m}^{phase}},m=1,2,\cdots ,p$$

The standardized test feature vector is $z_{\mathrm{new}}^{phase}={\left[z_{\mathrm{new},1}^{phase},\;z_{\mathrm{new},2}^{phase},\;\cdots ,\;z_{\mathrm{new},p}^{phase}\right]}^{T}$.

(3) Computation of the *T^2^* statistic for the test sortie

(11) $$T_{new,phase}^{2}=\sum_{m=1}^{p}\sum_{n=1}^{p}(z_{new,m}^{phase}-{\bar{z}}_{m}^{phase})a_{mn}^{phase}(z_{new,n}^{phase}-{\bar{z}}_{n}^{phase})$$

If $T_{new,phase}^{2}\leq UCL_{phase}$, the strain state of the wing in that sortie and flight phase is regarded as normal; otherwise, it is regarded as anomalous.

Once an anomaly is detected in a particular sortie and phase, a contribution plot is utilized to trace the dominant feature parameters by breaking down the *T*^2^ statistic into the contributions attributable to each original feature.

(4) Calculation of Contribution Value

The contribution $C_{m}$ of the *m*-th feature to the *T*^2^ statistic is defined as

(12) $$C_{m}=(Z_{new,m}^{phase}-{\bar{Z}}_{m}^{phase})\sum_{n=1}^{p}a_{mn}^{phase}(Z_{new,n}^{phase}-{\bar{Z}}_{n}^{phase}),(m=1,2,\cdots ,p)$$

It is noted that the contribution value $C_{m}$ of Equation (12) may be negative due to the multicollinearity among features. Specifically, since the inverse covariance matrix ${\left(S^{phase}\right)}^{-1}$ contains off-diagonal elements representing correlations between different features, a negative $C_{m}$ indicates that the deviation direction of the *m*-th feature is opposite to the weighted combination of other features, thereby partially “offsetting” the overall *T*^2^ statistic.

### 3.4. Assumption Assessment of Hotelling’s T^2^

To verify whether the baseline data satisfy the multivariate normality assumption required by the *F*-distribution-based control limit, Mardia’s test on the 30 normal flight sorties for each of the three flight phases (takeoff, cruise, and landing) was performed. The test results are summarized in Table 3. As shown in Table 3, all *p*-values are greater than 0.05, indicating that the assumption underlying the *F*-distribution-based control limit is considered acceptable.

## 4. Results and Discussion of Strain Data Characterization and Anomaly Identification

### 4.1. Results of Strain Data Characterization

It should be noted that the strain response of the wing can be influenced by operational and environmental factors, such as aircraft mass, flight speed, altitude, manoeuvres, and turbulence. In this study, the selected normal and test sorties were obtained from the same aircraft type under similar mission profiles. The takeoff weight, cruise altitude, and cruise speed were within comparable ranges, and no severe manoeuvres or strong turbulence were reported. Therefore, the operational variability among the selected sorties is considered limited. Furthermore, the phase-divided modeling strategy separates the three flight phases with distinct loading patterns, which reduces the influence of phase-related operational differences.

**Figure 5:**
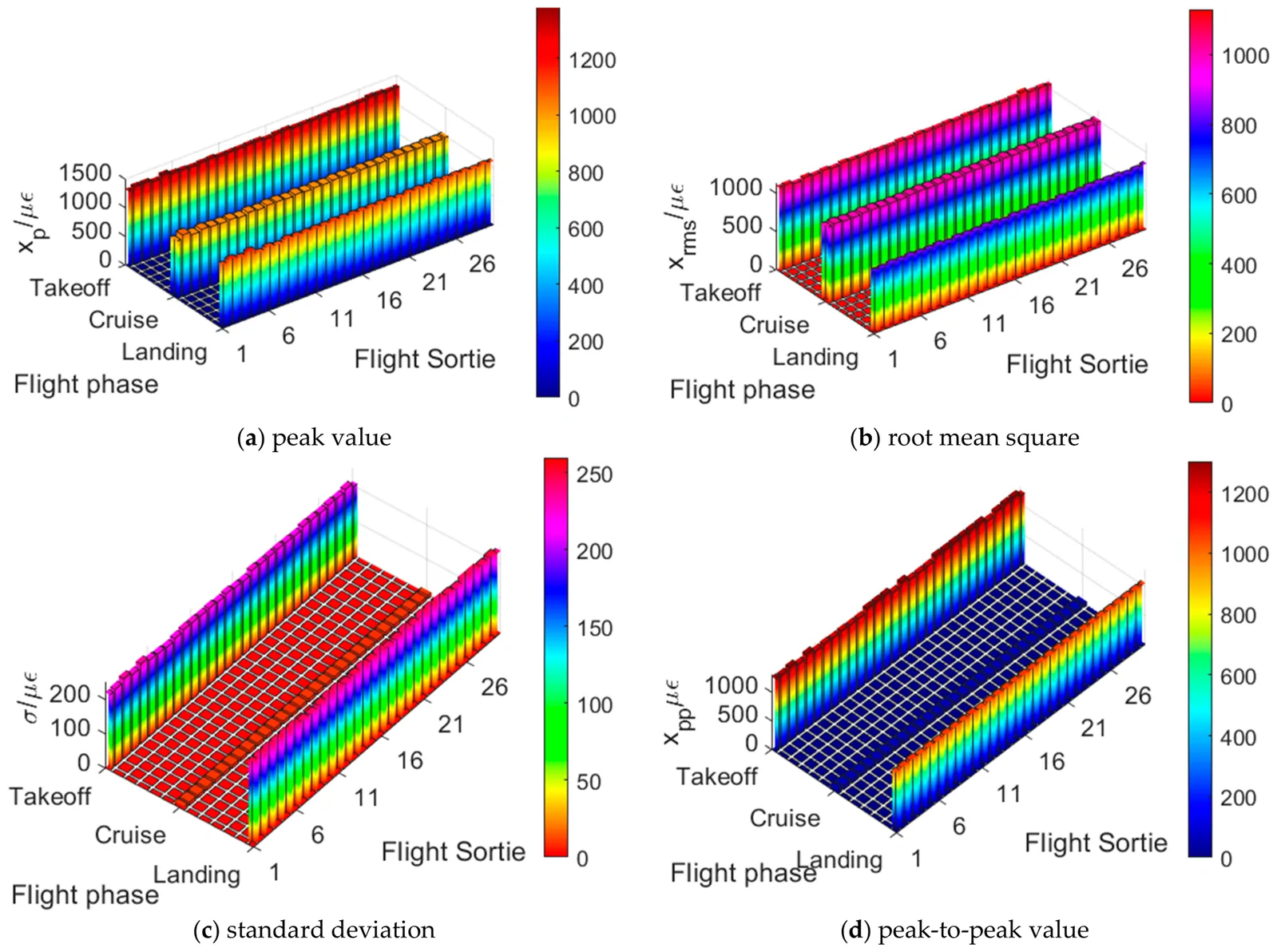

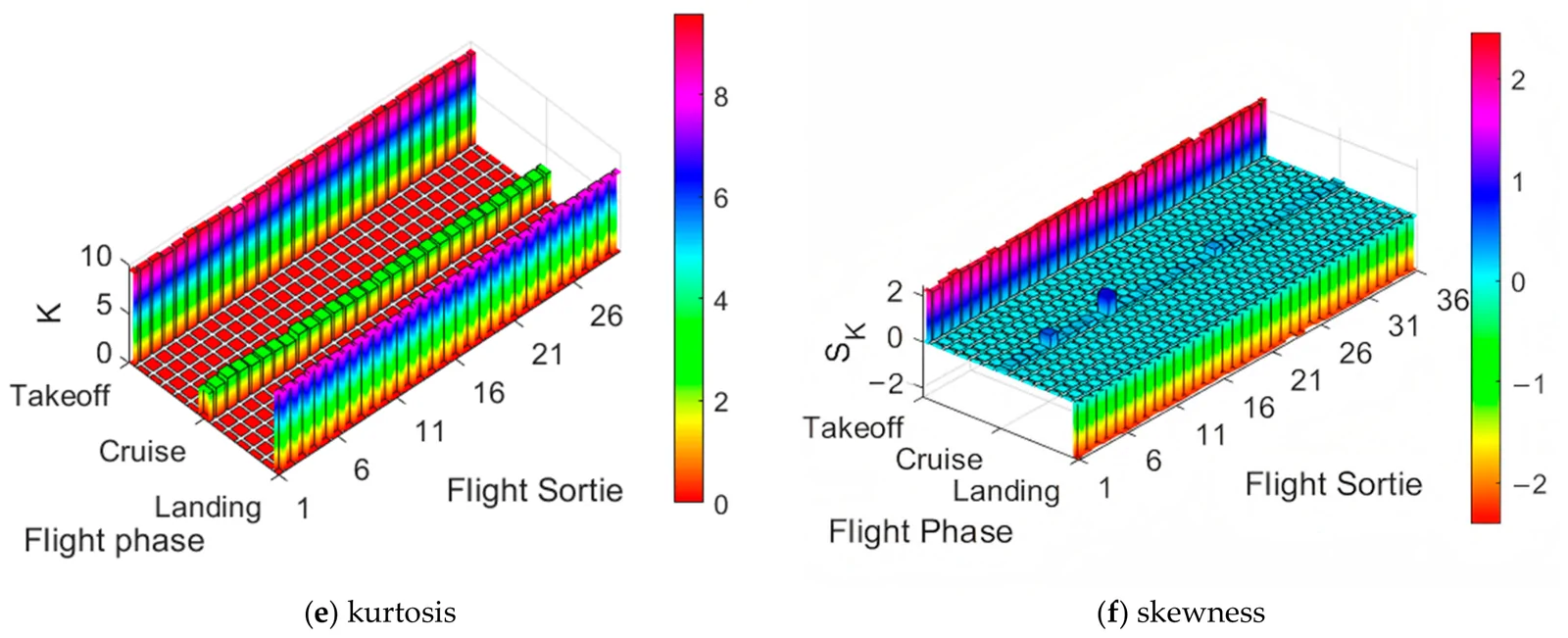
Time-domain strain features of the wing rear spar flange across different flight phases and sorties.

Figure 5 presents the statistical results of the six time-domain strain features of the wing rear spar flange across the three typical flight phases—takeoff, cruise, and landing—based on 30 normal flight sorties. The 30 baseline sorties were selected from flight records with no reported structural anomalies, no exceedance of operational limits, and no maintenance actions related to wing structural damage, thus representing normal operational conditions. The anomalous flight sorties described below were independently identified based on flight events documented in the flight logs (e.g., sudden turbulence encounters or maneuver overloads), which were absent in the baseline sorties.

As shown in Figure 5, the peak value is significantly higher during takeoff (approximately 1335 με) and landing (approximately 1129 με) than during the cruise phase (approximately 1032 με), corresponding to the tensile impact at liftoff and the load reversal upon touchdown, respectively. The RMS values during takeoff and cruise are comparable (approximately 1096 με and 1012 με, respectively), indicating that the wing continuously sustains high-level aerodynamic loads; the value during landing is the lowest (approximately 838 με), corresponding to lift unloading. The standard deviation is relatively large during takeoff and landing (approximately 221 με and 249 με, respectively), reflecting severe load fluctuations during these phases; it is minimal during cruise (approximately 10 με), indicating highly stable loading conditions. The peak-to-peak value during takeoff and landing (approximately 1269 με and 1021 με, respectively) fully captures the reversal process of load direction, while its value during cruise is the smallest (approximately 60 με). The kurtosis values corresponding to takeoff and landing phases reach approximately 9.4 and 8.1, respectively, both exceeding 3. This demonstrates that intense but normal impact loads emerge in these flight stages. During the cruise phase, the kurtosis is approximately 2.8, indicating the absence of remarkable abnormal disturbances. The skewness takes positive values during takeoff, indicating that tensile impact loads dominate, whereas it becomes negative during landing, reflecting the predominance of compressive loading.

In summary, the six feature parameters collectively characterize the wing strain state from multiple perspectives—maximum amplitude, energy level, fluctuation intensity, alternating range, impulsiveness and load direction—thereby providing a reliable baseline for subsequent anomaly identification.

To ensure the reliability of the multivariate statistical model, the correlations among the six features were examined. Table 4 summarizes the maximum correlation coefficients and the condition numbers of the covariance matrices for the three flight phases. The maximum correlation coefficients and condition numbers are all small, indicating that the covariance matrices are well conditioned and that multicollinearity is not a concern.

### 4.2. Results and Analysis of Anomaly Identification Diagnosis

**Figure 6:**
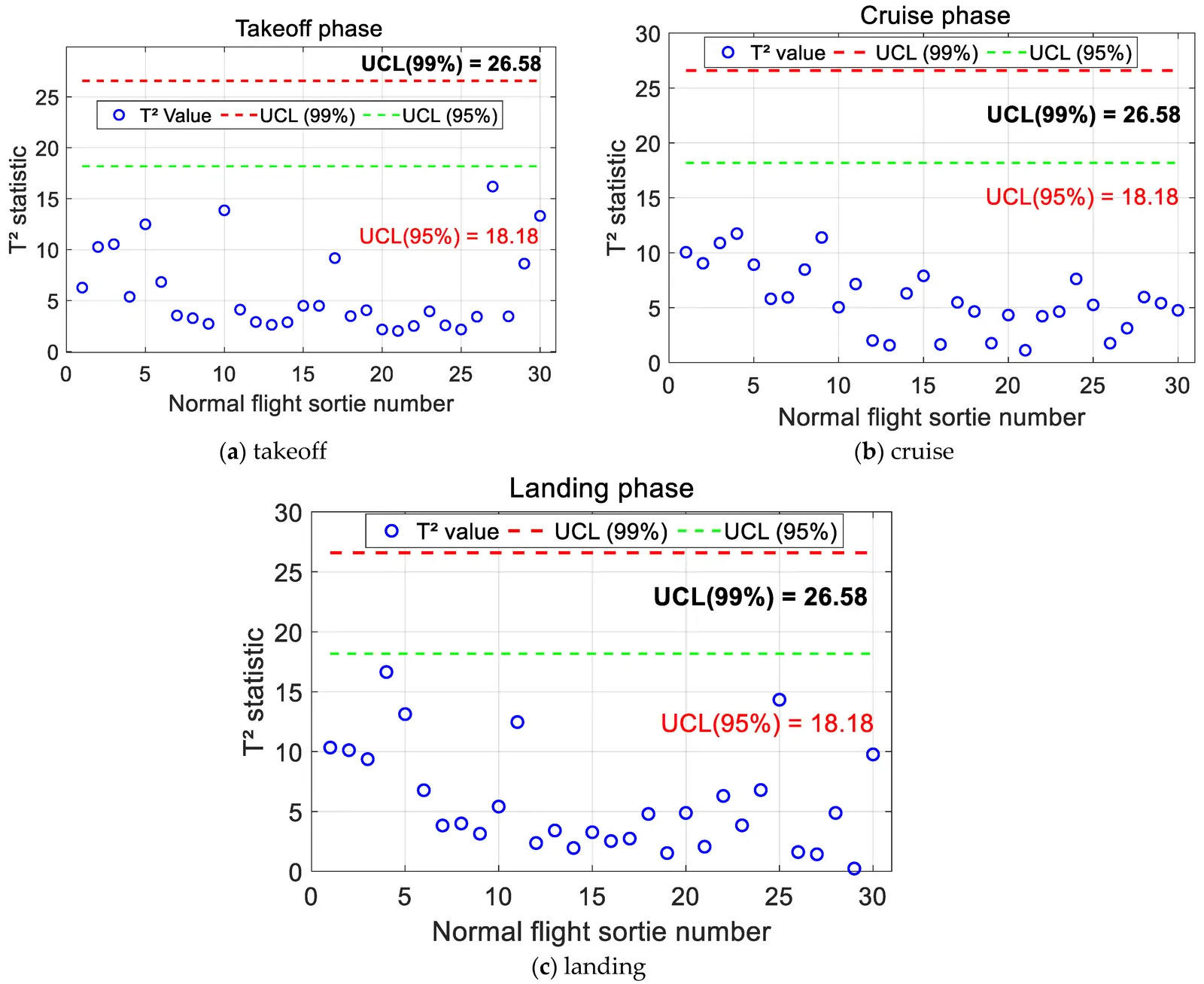
T^2^ control charts for normal flight sorties of the wing across takeoff, cruise, and landing phases.

Based on the six-dimensional time-domain feature vectors of 30 normal flight sorties in each flight phase, feature matrices of normal samples are separately established following the procedure in Section 3.2. The sample mean vector, covariance matrix, and $T^{2}$ statistic are subsequently calculated, and the upper control limit is determined as *UCL* = 26.58 at a 99% confidence level, as shown in Figure 6.

To validate the effectiveness of the proposed phase-divided MSPC method in wing strain data characterization and anomaly identification, anomaly diagnosis is performed on six test flight sorties of the wing structure (including three normal and three anomalous sorties) based on the established statistical control baseline for normal conditions. For each sortie, the six-dimensional time-domain feature vectors are extracted for the takeoff, cruise, and landing phases, respectively, and then fed into the corresponding statistical control model for each phase. The *T*^2^ statistic for each phase is computed and compared against the upper control limit.

**Figure 7:**
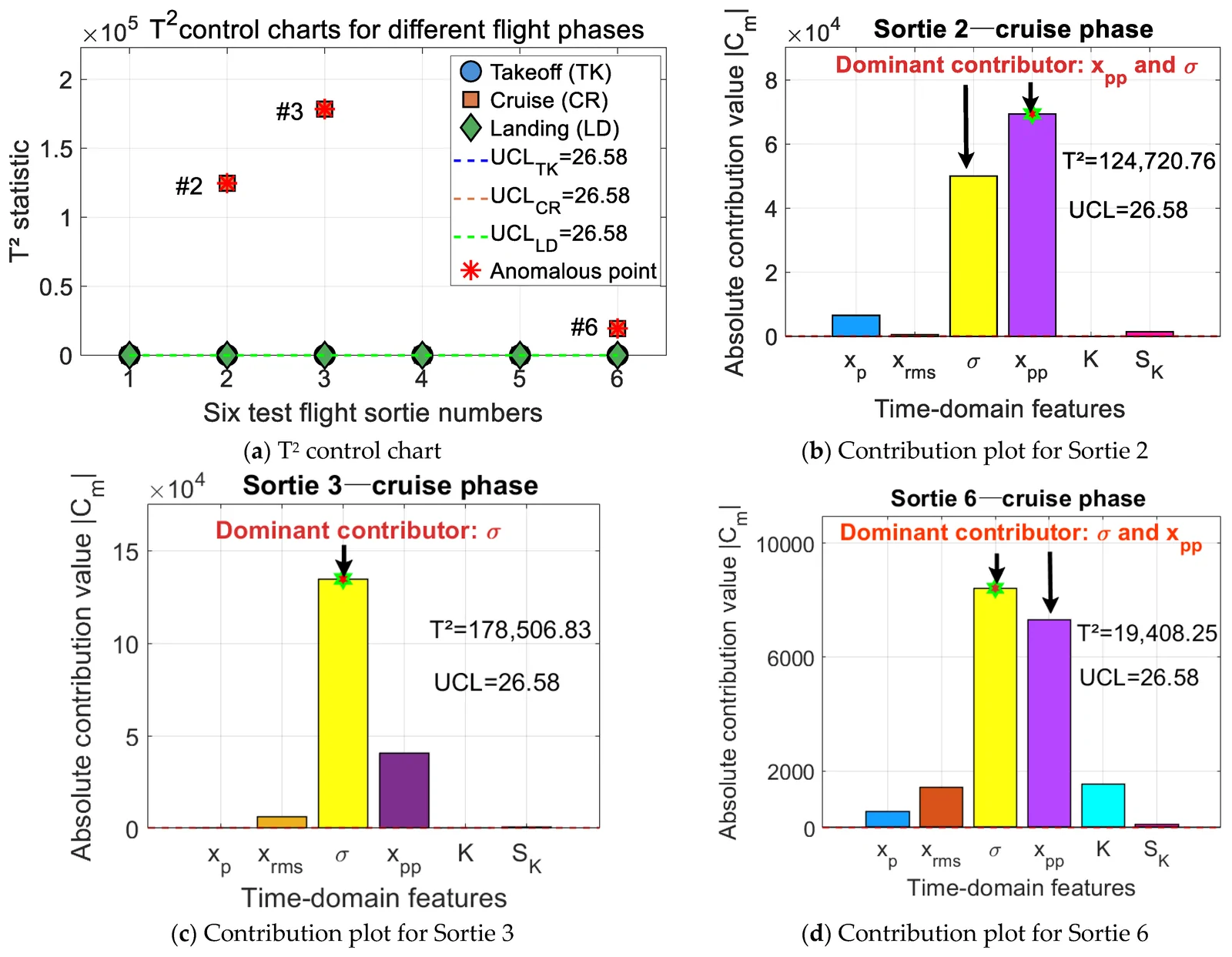
T^2^ control chart for six test flight sorties across different flight phases and contribution plots for anomalous sorties.

Figure 7 presents the *T*^2^ control charts for the six test sorties across different flight phases, along with the contribution plots for the anomalous sorties. Since the contribution values may be negative due to multicollinearity among features, the absolute values are used in the contribution plots for feature ranking. This ensures that the dominant features are correctly identified, while the signed contributions still sum to the *T*^2^ statistic. As can be seen from Figure 7a, the *T*^2^ values of sorties #1, #4, and #5 across all phases are below the control limit *UCL* = 26.58 at a 99% confidence level, and they are consequently classified as normal.

In contrast, sorties # 2, #3, and # 6 exhibit *T*^2^ values exceeding the control limit during the cruise phase and are identified as anomalous, indicating that the loading states of these sorties deviate from the normal baseline during the cruise phase. It is noted that the *T*^2^ values of the anomalous sorties are several orders of magnitude above the control limit (e.g., 124,720.76, 178,506.83, and 19,408.25). Such extremely large *T*^2^ values arise because the anomalous sorties exhibit multiple features that deviate by several standard deviations from the normal baseline. Since the *T*^2^ statistic is invariant to linear scaling (a property of the Mahalanobis distance), feature scaling and standardization do not affect the results. Therefore, these large values indicate exceptionally strong anomalies in the wing structural strain during the corresponding flight phases.

Figure 7b shows the contribution plot of Sortie #2 in the cruise phase. The $x_{pp}$ value and σ are the primary contributors to the anomaly, revealing that the alternating load range of this sortie is enlarged and the fluctuation intensity exceeds the normal threshold. Figure 7c presents the contribution plot for sortie #3 during the cruise phase, where the dominant contributor to the anomaly is identified as the σ, indicating an abnormal increase in the dispersion of loads during the cruise phase of this sortie. Figure 7d displays the contribution plot for sortie #6 in the cruise phase, with the σ and $x_{pp}$ value as the main contributors, revealing a combination of intensified load fluctuations and expanded alternating load range.

### 4.3. Quantitative Performance and Comparison

To quantitatively evaluate the stability of the proposed method, leave-one-out cross-validation (LOOCV) was performed on the 30 normal baseline sorties for each flight phase. This validation aims to assess the false alarm rate, i.e., the proportion of normal sorties that are incorrectly identified as anomalies. The results are summarized in Table 5. As shown in Table 5, the false alarm rates are 3.23% for takeoff, 0% for cruise, and 3.23% for landing. For both the takeoff and landing phases, only one out of the 30 normal sorties was incorrectly identified as an anomaly. The cruise phase achieved a 0% false alarm rate, indicating that the proposed model is highly stable under steady flight conditions. Additionally, a confusion matrix was constructed based on the six test sorties (three normal and three anomalous). The proposed method correctly identified all three anomalous sorties (sensitivity = 100%) and all three normal sorties (specificity = 100%), with a false-alarm rate of 0%. The accuracy and precision are also 100%. It should be noted that these metrics are based on a very small test set.

**Figure 8:**
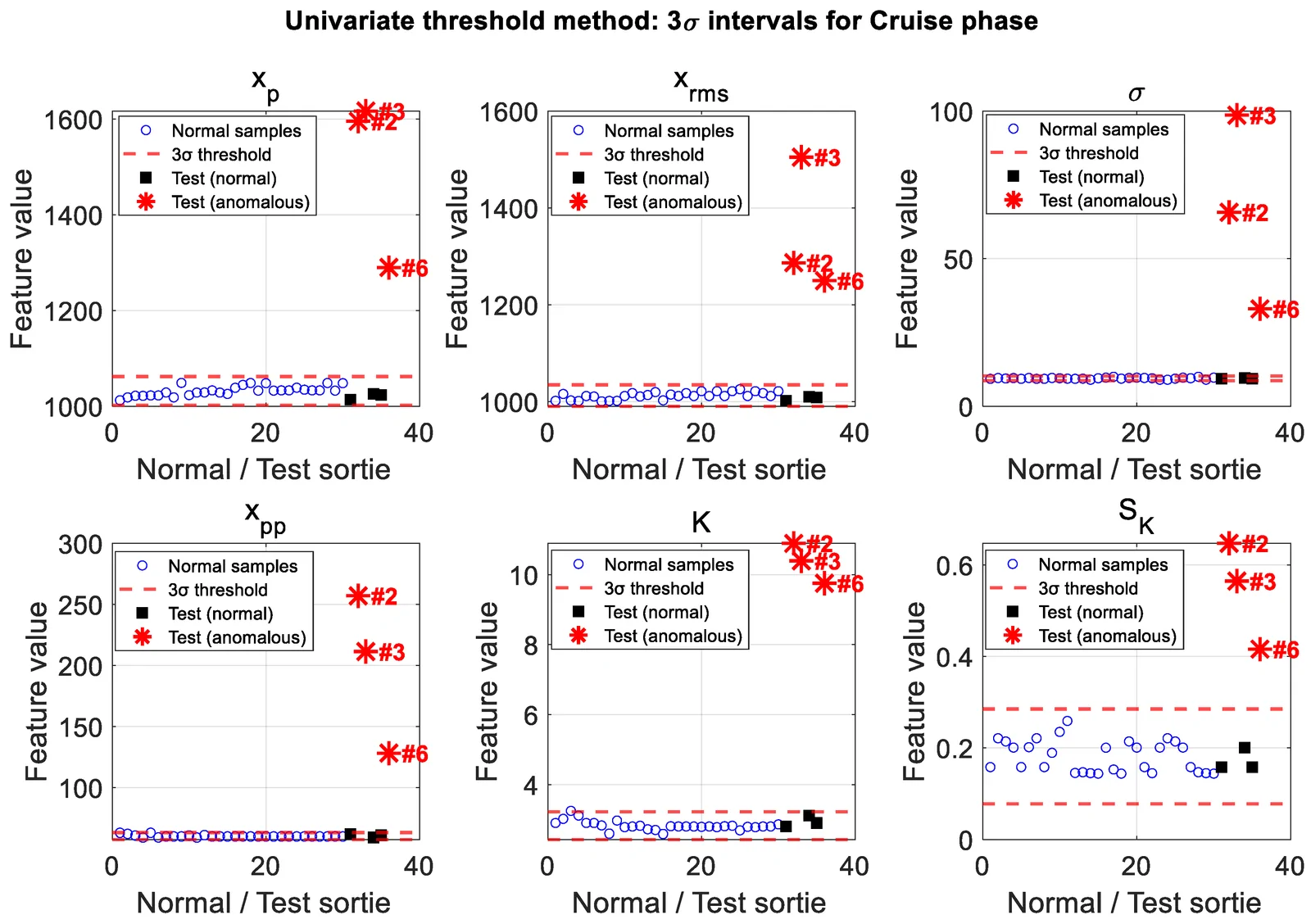
Univariate threshold method for the cruise phase.

To further evaluate the advantages of the proposed method, a univariate threshold method was implemented for comparison. Due to space limitations, only the result of the cruise phase is shown in Figure 8. As shown in Figure 8, although the univariate method can identify the anomalous sorties (#2, #3, and #6), it evaluates each feature independently and cannot capture synergistic anomalies among multiple features; moreover, a single feature occasionally exceeding the threshold may cause false alarms. In contrast, the proposed phase-divided MSPC method accounts for feature correlations and enables phase-specific anomaly detection and cause tracing.

## 5. Conclusions

Focusing on the rear spar flange of a specific aircraft wing, this paper proposes a phase-divided MSPC approach for strain data characterization and anomaly identification. Six time-domain features—peak value, RMS, standard deviation, peak-to-peak value, kurtosis, and skewness—are used to construct six-dimensional feature vectors for the takeoff, cruise, and landing phases. These features quantify the wing strain characteristics from six dimensions: amplitude limit, alternating range, energy level, dispersion, impact intensity, and load direction. Combined with Hotelling’s $T^{2}$ control charts and contribution plots, the method achieves quantitative characterization and anomaly diagnosis of wing strain states. Based on the control limit UCL = 26.58 determined from 30 normal sorties at a 99% confidence level, diagnosis of six test sorties identifies three sorties with anomalous strain patterns (all in the cruise phase), and their dominant features are traced via contribution plots, demonstrating engineering applicability. Compared with deep learning methods such as WSAT, the proposed method offers advantages in small-sample capability and statistical interpretability, but its ability to capture highly nonlinear relationships remains limited. Future work will validate the method with larger datasets and different operating conditions, and will employ independent verification (e.g., non-destructive inspection) to confirm whether the detected anomalous strain patterns correspond to actual structural anomalies.

## Figures and Tables

**Table 1:** Relevant information on the FBG sensors and the data acquisition and recording system.

**FBG Type**	**Sampling Frequency**	**Sensor Installation Location**
Substrate-type FBG strain sensor	100 Hz	Rear spar flange of wing
**Strain Unit**	**Data Acquisition Model**	**Data Recorder Model**
με	XMA	MDR

**Table 2:** Definitions of the six typical time-domain statistical feature parameters.

Parameter	Definition	Parameter	Definition
peak value	$x_{p}=max(x_{i})$	peak-to-peak value	$x_{pp}=\max(x_{i})-\min(x_{i})$
root mean square	$x_{rms}=\sqrt{\frac{1}{N}\sum_{i=1}^{N}{x_{i}}^{2}}$	kurtosis	$K=\frac{\frac{1}{N}\sum_{i=1}^{N}{\left(x_{i}-\bar{x}\right)}^{4}}{\sigma^{4}}$
standard deviation	$\sigma =\sqrt{\frac{1}{N}\sum_{i=1}^{N}{\left(x_{i}-\bar{x}\right)}^{2}}$	skewness	$S_{K}=\frac{\frac{1}{N}\sum_{i=1}^{N}{\left(x_{i}-\bar{x}\right)}^{3}}{\sigma^{3}}$

Note: $x_{i}$ is the time-domain signal sequence, where i=1,2,⋯,N and *N* is the number of sampling points.

**Table 3:** *p*-values of skewness and kurtosis tests for different flight phases.

*p*-Values for Different Flight Phases	Takeoff	Cruise	Landing
*p*-value of skewness	0.069	0.137	0.073
*p*-value of kurtosis	0.67	0.129	0.317

**Table 4:** Feature correlation and covariance matrix conditioning for different flight phases.

Flight Phase	Takeoff	Cruise	Landing
**Maximum Correlation Coefficient**	0.502 (Peak and Std)	0.430 (Peak and RMS)	0.495 (Peak-to-Peak and Skewness)
**Condition Number**	6.48	7.37	3.69

**Table 5:** False alarm rates of the proposed method for different flight phases based on LOOCV.

Flight Phase	Total Held-Out Sorties	Number of False Alarms	False Alarm Rate
Takeoff	30	1	3.23%
Cruise	30	0	0
Landing	30	1	3.23%

## Data Availability

The data presented in this study are available on request from the corresponding author.
